# Comparison of NUCLEOCOUNTER, ANDROVISION with Leja chambers and the newly developed ANDROVISION eFlow for sperm concentration analysis in boars

**DOI:** 10.1038/s41598-022-16280-6

**Published:** 2022-07-13

**Authors:** Rudolf Grossfeld, Julia Pable, Ulrike Jakop, Christian Simmet, Martin Schulze

**Affiliations:** 1 Minitüb GmbH, Hauptstraße 41, 84184 Tiefenbach, Germany; 2 Institute for Reproduction of Farm Animals Schönow, Bernauer Allee 10, 16321 Bernau, Germany

**Keywords:** Biotechnology, Cell biology, Zoology

## Abstract

Exact analysis of sperm concentration in raw and diluted semen is of major importance in swine artificial insemination, as sperm concentration is one of the most important characteristics of an ejaculate determining the value of the ejaculate and the productive life of the boar. The study compares different methods for sperm concentration analysis in raw and diluted boar semen: NUCLEOCOUNTER SP-100, the ANDROVISION with Leja chambers and the new ANDROVISION eFlow system. The Concordance Correlation Coefficient (CCC) between NUCLEOCOUNTER and ANDROVISION eFlow was 0.955 for raw (n = 185 ejaculates) and 0.94 for diluted semen (n = 109 ejaculates). The CCC between NUCLEOCOUNTER and ANDROVISION with Leja chambers was 0.66. A Bland–Altman plot of split-sample measurements of sperm concentration with NUCLEOCOUNTER and ANDROVISION eFlow showed that 95.1% of all measurements lay within ± 1.96 standard deviation. The coefficients of variance were 1.6 ± 1.3%, 3.6 ± 3.6% and 7.3 ± 6.3% for NUCLEOCOUNTER, ANDROVISION eFlow and ANDROVISION with Leja chambers in diluted semen, respectively. NUCLEOCOUNTER and ANDROVISION eFlow are comparable tools to measure the concentration of raw and diluted boar semen. In comparison to ANDROVISION with Leja chambers, concentration analyses of diluted semen using NUCLEOCOUNTER or ANDROVISION eFlow show a higher repeatability within and a higher concordance between the methods.

## Introduction

The determination of sperm concentration is an important part of the quality control of raw boar ejaculates during sperm production in every artificial insemination (AI) center. The correct evaluation of boar sperm concentration in raw semen as well as diluted semen in the doses is vital for highest efficacy in AI. A uniform insemination dose becomes more important as AI centers are aiming at reducing the number of sperm per AI dose^1,2^. The accuracy and precision of the sperm concentration measurement of a boar ejaculate has a direct influence on the number of producible sperm doses from a particular boar ejaculate. Moreover, the number of producible sperm doses is directly related to the monetary turnover, as sperm doses are usually a significant source of income for an AI center. The higher the genetic value of the boar, the more important the number of producible sperm doses becomes^3^.

There are different methods for the analysis of sperm concentration^4^. The oldest and most basic method is to use a counting chamber (hemocytometer) and manually count the sperm cells in a grid with a defined volume under a microscope, preferentially with phase contrast. If performed by trained staff, this method generates reliable results but it is rather time consuming and therefore inefficient for general use in AI centers^5,6^. Due to time constraints measurement with a photometer is more suitable, where sperm concentration is determined by the optical density of the ejaculate^7^. This was the method of choice several years ago in AI centers, but since analysis of the optical density is relatively imprecise, it is increasingly being replaced by other techniques.

The sperm concentration can be analyzed precisely and relatively fast with a NUCLEOCOUNTER SP-100 (ChemoMetec A/S, Allerod, Denmark). As the NUCLEOCOUNTER generates reliable results, it can be used as a reference method^8,9^. Disadvantages are the relatively high cost of the device and consumables. A flow cytometer or FACS (Fluorescence Activated Cell Sorting) is also useful in determining the concentration of sperm^10^. However, the high acquisition costs can only be justified if the flow cytometer is also used for physiological analyses of the sperm, which is currently only possible for very large, selected AI centers or reference laboratories^5^.

AI centers increasingly use computer assisted sperm analysis (CASA) systems for sperm concentration analysis^11^, as concentration can be analyzed simultaneously with sperm motility and basic morphology. CASA systems require standardized counting chambers in order to determine the sperm concentration. Common counting chambers are disposable and consist of a glass slide and a cover-slip with a defined chamber height^12^, thus allowing the determination of sperm concentration in the sample. Precision of measurement highly depends on exact pipetting volumes, training of lab personnel and especially a consistent quality of the chambers among product batches^4,13^. There are also reusable chambers with a defined chamber height available, e.g., the MAKLER counting chamber (Sefi-Medical Instruments, Haifa, Israel), which offer a high accuracy if used correctly by skilled personal^14,15^. But the time required for cleaning is too high for use in AI centers.

A new method for sperm concentration analysis with a CASA system is the ANDROVISION eFlow system (Minitüb, Tiefenbach, Germany), a reusable chamber combined with a fluid management system (ANDROVISION eFlow system, US Patent 10 768 087) that enables the exact determination of sperm concentration during motility assessment. The chamber is filled and flushed between samples by a fluid management system (FMS) and is automatically closed during analysis to enable measurement in a closed system with defined volume. The high degree of automation decreases the influence of the user during the process of sperm concentration measurement, which could have a significant effect on the measurement result^13^. In this study, the accuracy and usability of the ANDROVISION eFlow system in determining sperm concentration of raw and diluted boar semen was compared to established methods, i.e., the NUCLEOCOUNTER SP-100 and a disposable counting chamber with CASA.

## Material and methods

### Experimental design for comparison of sperm concentration measurement

To validate the precision and correctness of sperm concentration measurement in boar ejaculates with the ANDROVISION eFlow system, two trials were conducted. In experiment 1**,** the concentration of raw semen (n = 185 boar ejaculates) was determined in split-samples by the ANDROVISION eFlow system and the NUCLEOCOUNTER SP-100 as a reference method and the concordance of both methods was analyzed.

In experiment 2, the sperm concentration of diluted boar semen (n = 109 boar ejaculates) was determined in split-samples by the ANDROVISION eFlow system, ANDROVISION with disposable counting chambers (Leja, 4-chamber counting slides; 20 µm nominal chamber depth, Leja Products B.V., Nieuw-Vennep, The Netherlands) and the NUCLEOCOUNTER SP-100 as a reference method^8,9^. The concordance and repeatability for sperm concentration assessment with all three methods were analyzed.

### Animals, semen collection and semen processing

All procedures involving animals were carried out in accordance with guidelines and regulations according to the European Commission Directive for Pig Welfare and follows the recommendations in the ARRIVE guidelines. The experimental protocol was approved by the institutional animal welfare committee of the IFN (Reg. 2021/02). Present study is not an animal experiment. Ejaculates were collected weekly. The boars (mean age: 1.91 ± 1.2 years) received commercial feed (pellets) for AI boars and were housed individually in straw-bedded pens equipped with nipple drinkers. Semen processing protocols followed the general guidelines of the Institute for Reproduction of Farm Animals Schönow (IFN) for semen production^16^. Ejaculates were collected by the double gloved-hand method on a routine basis in a pre-warmed (40 °C) glass container (500 mL) with an insulated cover cup. The pre-sperm phase of each ejaculate was discarded and the gel fraction of the semen was removed by gauze filtration during collection. After collection of the sperm rich and sperm poor fraction, the glass container was closed and transported to the laboratory with a pneumatic system. The protocols for conducting the measurements with all three methods followed the recommendations in the supplement to Brito et al*.*^4^.

#### Experiment 1: Comparison of NUCLEOCOUNTER and ANDROVISION eFlow for concentration analysis in raw semen samples

To compare both methods, the sperm concentration of 185 raw boar ejaculates was measured in split-samples with both, the NUCLEOCOUNTER (SP-100, ChemoMetec A/S, Allerod, Denmark) and ANDROVISION eFlow (Minitüb GmbH, Tiefenbach, Germany). Upon receiving the samples for sperm concentration, measurement with the NUCLEOCOUNTER and ANDROVISION eFlow were taken directly from the same raw ejaculate (split-samples) and analyzed immediately.

##### Evaluation of sperm concentration in raw semen with NUCLEOCOUNTER SP-100

After careful homogenization of the ejaculate by turning the glass container overhead five times, a sample of 50 µL raw semen were pipetted from the middle of the glass container with an immersion depth of 1 cm. The sample was then transferred into a sample cup (ChemoMetec A/S, Allerod, Denmark). The sample was diluted 1 + 100 (v/v) with 5 mL Reagent S100 (ChemoMetec) using a Dispensette III (Brand GmbH, Wertheim, Germany), according to the manufacturer's recommendations. Adding of Reagent S100 assured a sufficient mixing of the sample. Immediately thereafter, a sample of the diluted semen was aspirated into the SP1 Cassette (ChemoMetec), and analyzed in the NUCLEOCOUNTER. All NUCLEOCOUNTER measurements were performed as double measurements. The mean of each double measurement was used as the reference value for sperm concentration of each boar ejaculate.

##### Evaluation of sperm concentration in raw semen with ANDROVISION eFlow

**Figure 1:**
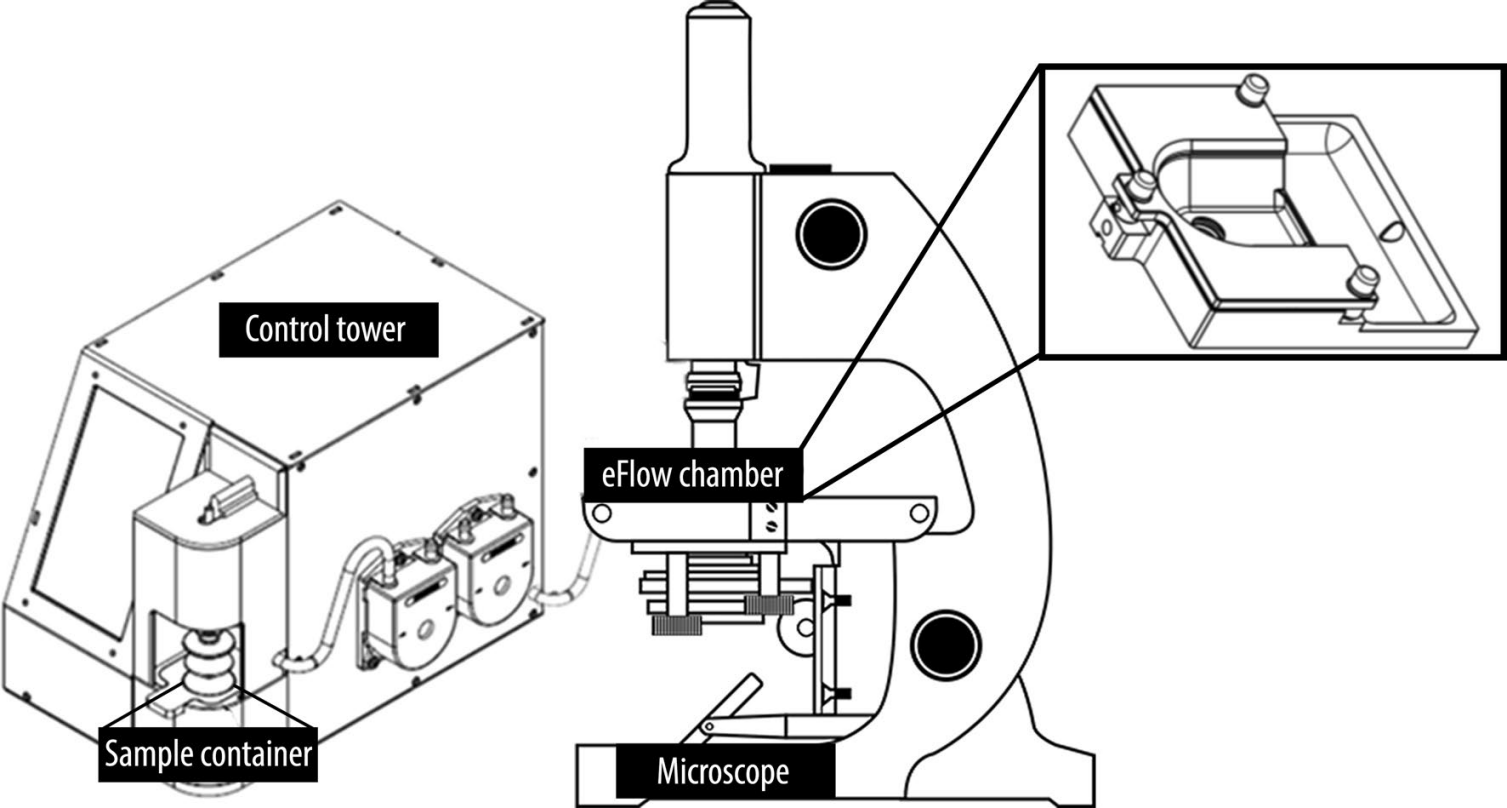
Overview of control tower with fluid management system and the eFlow chamber on a microscope stage. The pre-diluted sperm sample is automatically transferred from the sample container to the reusable eFlow chamber under the microscope objective. The microscope has a camera, which is connected to a PC with the ANDROVISION CASA software.

For the measurement of the sperm concentration with ANDROVISION eFlow, 7.2 mL of BTS boar sperm extender without antibiotics (Minitüb) was used to pre-fill a sample container (Minitüb) with a Dispensette III (Brand GmbH). Thereafter, 600 µL raw semen were taken from the middle of the glass container with an immersion depth of 1 cm (E4-2000XLS Pipette, Mettler-Toledo, Gießen, Germany) and added to the pre-filled sample container (dilution rate: 1 + 12 (v/v)), in order to reach the optimal range for sperm concentration analysis (150–550 sperm per analysis field), as recommended by the manufacturer. After that, the sample container was closed with a rubber plug and carefully turned overhead minimum 5 times to homogenize the diluted semen. The sample container was then put into the fluid-management system (FMS) and the sperm concentration was analyzed with the ANDROVISION CASA. The FMS of ANDROVISION eFlow automatically transferred the pre-diluted sperm sample in a closed system into the eFlow chamber by quenching the foldable sample container and simultaneously taking up the sample in a silicone tube (inner diameter 3 mm, length 23 cm), which is connected to the eFlow chamber (Fig. 1). By this, the sperm sample is flushed into the measurement area of the chamber. The eFlow counting chamber includes a mechanism that allows to open the measurement gap to 500 µm in order to flush the sample into the measurement area. After filling the mechanism, closes the measurement gap to the preadjusted gap heights. The measurement area of the chamber consists of two parts, one with a height of 15 µm and a second part with a height of 30 µm. Sperm concentration was measured in the part with 30 µm height. The chamber height was controlled prior to performing the eFlow analysis with the gap measurement tool which is a photometry based gap measurement included in the eFlow system, in order to assure the correct chamber height.

For determining the sperm concentration, the software ANDROVISION (Version 1.2) was used. The videos of the sperm samples in the eFlow were acquired with an Axioscope A1 (Carl Zeiss Microscopy GmbH, Oberkochen, Germany) and 10 × negative phase contrast objective. The microscope was connected to a Basler camera (Aviator avA1000-100gm, Basler AG, Ahrensburg, Germany), which was connected to the ANDROVISION-PC.

During the measurement procedure, three measurement fields on the middle axis in each part of the eFlow chamber (15 µm and 30 µm gap) were accessed by means of an automatic scan stage that was mounted under the microscope and automatically controlled by the ANDROVISION software without user influence. Videos were recorded with a resolution of 0.3 megapixel and a frame rate of 60 Hz for 0.5 s. The recorded measurement area in each measurement field was 512 × 512 µm. For sperm concentration measurement, three fields were recorded in the 30 µm gap area.

##### Experiment 2: Comparison of NUCLEOCOUNTER, ANDROVISION eFlow and ANDROVISION with Leja chambers for concentration analysis in diluted semen samples

To compare the concentration measurements of different methods, 109 diluted boar semen samples from a commercial AI center were measured for sperm concentration with the NUCLEOCOUNTER, ANDROVISION eFlow and ANDROVISION with disposable counting chambers (Leja, 4-chamber counting slides; 20 µm nominal chamber depth, Leja Products B.V., Nieuw-Vennep, The Netherlands) in a split sample design. The semen tubes were randomly taken during the daily production of the AI center. The target sperm content per semen tube was 1.8 billion sperm. All boar semen samples were tested in double measurements and the coefficient of variation (CV) was calculated for each sample and each method. All semen samples were obtained in 85 mL Quicktip Flexitubes (Minitüb) and pre-warmed to 38 °C for at least 15 min before analysis. All semen samples were taken from exactly the same semen tube (sample set) for each measurement system in parallel.

##### Evaluation of sperm concentration in diluted semen with NUCLEOCOUNTER SP-100

After careful homogenization of the sperm dose (volume: 85 mL) by turning the closed sperm dose overhead five times, 500 µL diluted sperm were pipetted (E4-2000XLS Pipette, Mettler-Toledo, Gießen, Germany) into a sample cup (ChemoMetec, A/S, Allerod, Denmark). The sample was diluted 1 + 10 with 5 mL Reagent S100 (ChemoMetec) using a Dispensette III (Brand GmbH), according to the manufacturer's recommendations. Adding Reagent S100 assured sufficient mixing of the sample. Immediately thereafter, a sample of the diluted semen was aspirated into the SP1 Cassette (ChemoMetec) and analyzed in the NUCLEOCOUNTER as described by the manufacturer.

##### Evaluation of sperm concentration in diluted semen with ANDROVISION eFlow

For measuring sperm concentration with ANDROVISION eFlow and after careful homogenization of the sperm dose, 6–8 mL diluted semen were transferred into the eFlow sample container (Minitüb), as the expected sperm concentration lied within the range that is recommended by the manufacturer (150–550 sperm per analysis field). The sample container was then put into the FMS without further dilution and the sperm concentration was analyzed with the ANDROVISION CASA, as described in chapter "Evaluation of sperm concentration in raw semen with ANDROVISION eFlow". For the sperm concentration determination, ANDROVISION (Version 1.2) was used. The videos of the sperm samples in the eFlow were acquired with an Axioscope A1 (Carl Zeiss Microscopy GmbH, Oberkochen, Germany) and 10 × negative phase contrast objective. The microscope was connected to a Basler camera (acA2440-75uc, Basler AG, Ahrensburg, Germany), which was connected to the ANDROVISION-PC. During the measurement procedure, three measurement fields on the middle axis in each part of the eFlow chamber (15 µm and 30 µm gap) were accessed by means of an automatic scan stage that was mounted under the microscope and controlled by the ANDROVISION software. Videos were recorded with a resolution of 0.5 megapixel and a frame rate of 60 Hz for 0.5 s. The recorded measurement area in each measurement field was 706 × 706 µm. For sperm concentration measurement three fields were recorded in the 30 µm gap.

##### Evaluation of sperm concentration in diluted semen with ANDROVISION and Leja chamber

After careful homogenization of the sperm dose, 2.7 µL of diluted semen sample were loaded into a pre-heated Leja chamber using a 10 µL pipette (Pipet Lite L 10 XLS, Mettler Toledo, Gießen, Germany) without further dilution, as the expected sperm concentration lied within the range, as recommended by the manufacturer of the CASA system (80–300 sperm per analysis field). The chamber was placed on a heated microscope stage on an Axioscope A1 (Carl Zeiss Microscopy GmbH) with an automated scan stage (Minitüb). The sperm concentration was analyzed directly thereafter with the ANDROVISION CASA. The CASA system automatically chose six measurement fields in the middle axis of each chamber of the slide and averaged the results from these fields. The CASA software was connected to an automatic scan stage under the microscope that allowed a repeatable access of the measurement areas in each slide without user influence. For the analysis with the Leja chamber, the same microscope and camera were used as described in chapter "Evaluation of sperm concentration in diluted semen with ANDROVISION eFlow". Videos were recorded with a resolution of 0.5 megapixel and a frame rate of 60 Hz for 0.5 s. The recorded measurement area in each measurement field was 706 µm × 706 µm, as well.

### Statistical analysis

The statistical analysis was performed with R 4.0.3^17^. Values are expressed as mean ± standard deviation (SD). For the evaluation of the method-agreement of sperm concentration measurements the Concordance Correlation Coefficient (CCC) was calculated^18,19^. The measurement results per method were additionally compared with a two-sample Student’s *t*-test for paired samples. The Bland–Altman plot was used to visualize the agreement of the results of the two methods^20^. The repeatability of each instrument was evaluated by determining the coefficient of variance (CV). The respective CV values for each sample in each measurement method were compared with an analysis of variance. Tukey’s Honest Significant Differences were calculated for multiple comparisons. The Friedman rank sum test was applied to compare the means of double measurement per each sample concentration to test if the results of the respective evaluation methods differed significantly. The Wilcoxon signed-rank test with Bonferroni correction was then used for multiple pairwise comparisons. Values were considered significantly different if the *P*-value was ≤ 0.05.

## Results

### Experiment 1: Method comparison of concentration analysis in raw semen with NUCLEOCOUNTER and ANDROVISION eFlow

**Figure 2:**
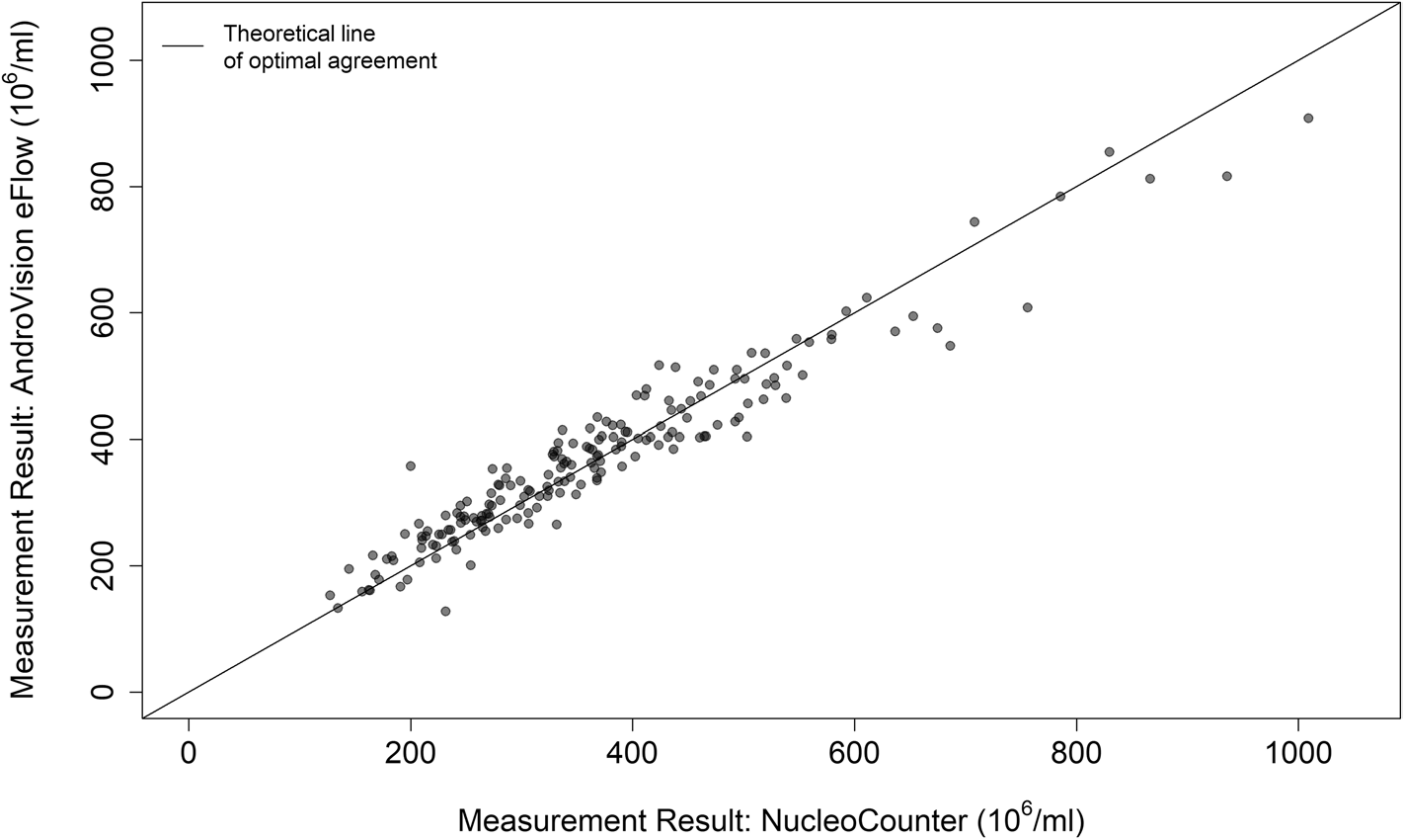
Method comparison of NUCLEOCOUNTER and eFlow sperm concentration of 185 raw boar ejaculates as measured in split samples with the NUCLEOCOUNTER as reference method and ANDROVISION eFlow. The Concordance Correlation Coefficient (CCC) of the method comparison was 0.955.

**Figure 3:**
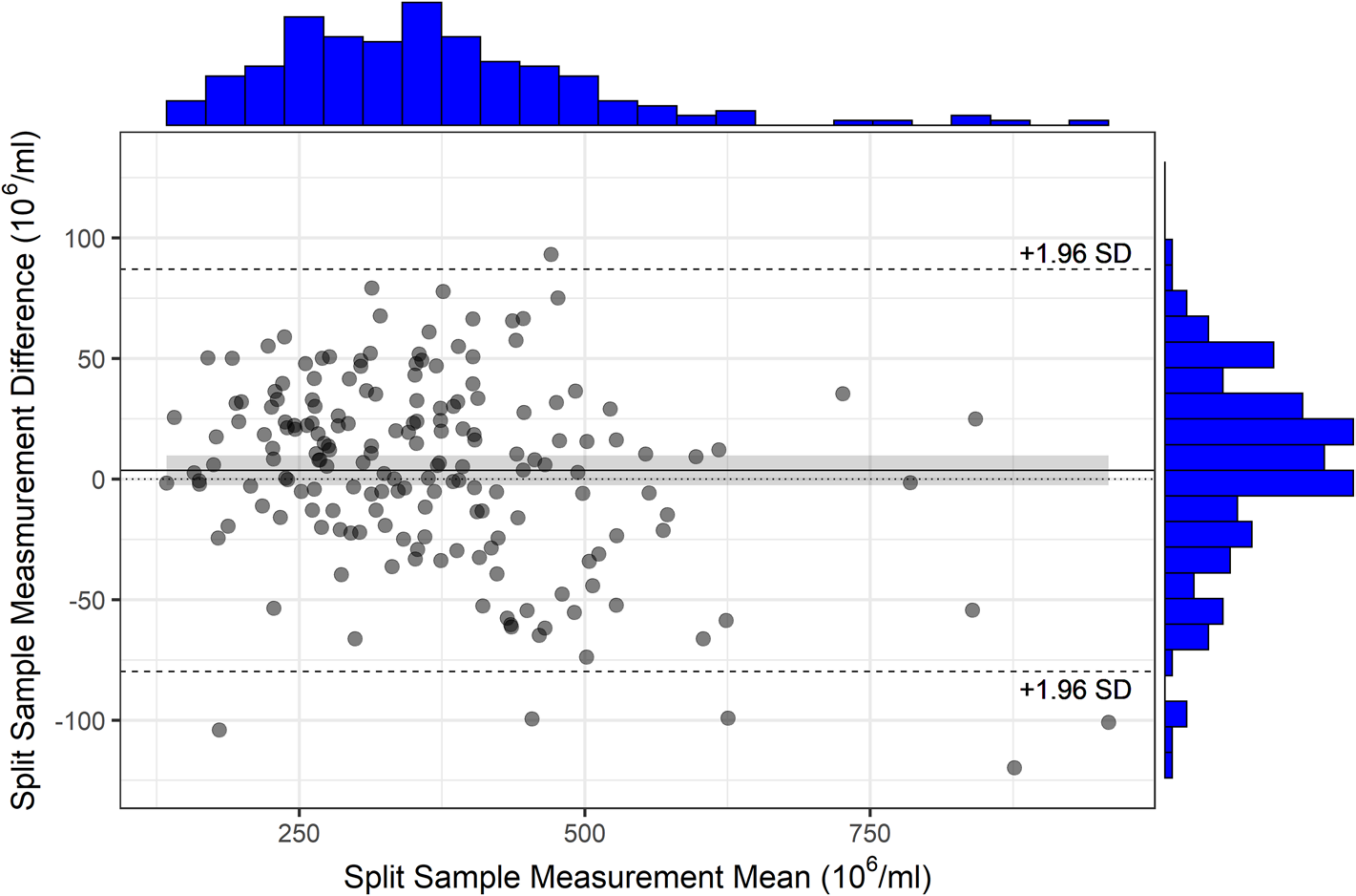
Bland–Altman plot of split-sample measurements of sperm concentration with ANDROVISION eFlow and NUCLEOCOUNTER (reference method). 95.1% of all measurements lie within mean ± 1.96 SD. The confidence interval (shaded area) of the mean of the absolute differences (± 6.13 × 10^6^, CI =  − 2.51 × 10^6^–9.75 × 10^6^) includes the x-axis.

In total, 185 raw boar ejaculates were analyzed for their sperm concentration with both the NUCLEOCOUNTER and ANDROVISION eFlow. The mean value for each sample did not differ significantly between the two methods (paired Student’s *t*-test; *P* = 0.25, Table 1). A Concordance Correlation Coefficient (CCC) between NUCLEOCOUNTER and ANDROVISION eFlow could be calculated with 0.955 (Fig. 2), which corresponds to a nearly complete concordance of the measurement results of ANDROVISION eFlow and the NUCLEOCOUNTER as a reference method^21^. Figure 3 shows a Bland–Altman plot of the split-sample measurements with both methods. 95.1% of all measurements lie within ± 1.96 SD.

**Table 1:** Results (mean, SD, minimum and maximum) of sperm concentration measurement with two methods (NUCLEOCOUNTER, ANDROVISION eFlow) from 185 raw boar ejaculates. The mean values for sperm concentration did not differ significantly (paired Student’s t-test; *P* = 0.25).

Numbers	NUCLEOCOUNTER (sperm/mL)	ANDROVISION eFlow (sperm/mL)
Mean	366.9 × 10^6^	370.6 × 10^6^
SD	150.8 × 10^6^	135.0 × 10^6^
Min	127.6 × 10^6^	127.8 × 10^6^
Max	1009.0 × 10^6^	908.1 × 10^6^

### Experiment 2: Method comparison of sperm concentration analysis in diluted semen with NUCLEOCOUNTER, ANDROVISION eFlow and ANDROVISION with Leja chambers as well as repeatability within each method

**Figure 4:**
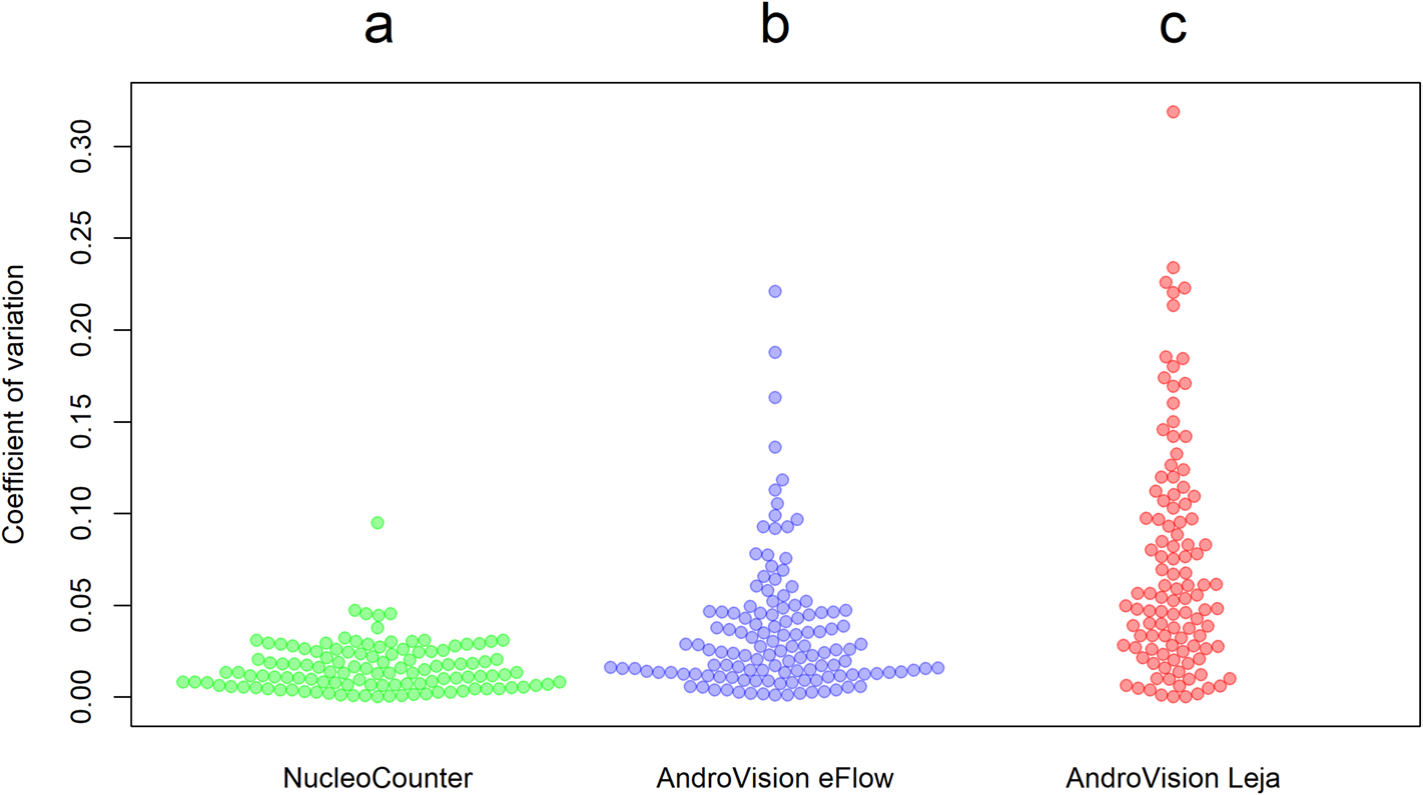
Plots of coefficient of variation (CV) of sperm concentration double measurements with NUCLEOCOUNTER, ANDROVISION eFlow and Leja chamber, respectively (^a,b,c^ *P* < 0.0001).

**Figure 5:**
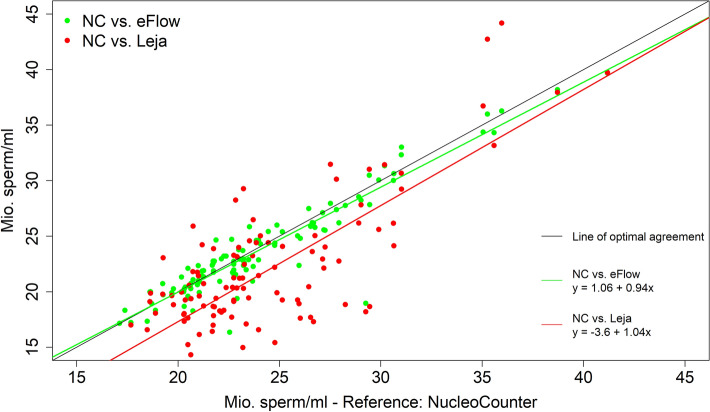
Comparison of boar sperm concentrations (diluted semen) measurements methods. ANDROVISION eFlow and disposable counting chambers (Leja) were tested against the NUCLEOCOUNTER (NC), respectively. The linear regressions are defined as y = 1.06 + 0.94 × for ANDROVISION eFlow and y =  − 3.6 + 1.04 × for ANDROVISION with Leja chambers.

The mean coefficients of variation (CV) for double measurements of 109 diluted boar sperm samples are summarized in Fig. 4 for all three measurement methods. The mean CV for repeated measurements of split sample boar ejaculates was 1.6 ± 1.3%, 3.6 ± 3.6% and 7.3 ± 6.3% for the NUCLEOCOUNTER, ANDROVISION eFlow and the ANDROVISION with Leja chamber, respectively. The CVs for all measurement values are significantly different against each other (*P* < 0.0001). Figure 5 demonstrates the correlation of the measurement *versus* ANDROVISION with Leja chambers, respectively. The CCC was 0.94 for the NUCLEOCOUNTER and ANDROVISION eFlow results. The CCC for the NUCLEOCOUNTER and ANDROVISION with Leja chamber sperm concentration results was 0.66, which implies strong concordance^21^. The Friedman rank sum test and Wilcoxon test for pairwise comparisons revealed no significant difference between the results of the NUCLEOCOUNTER and ANDROVISION eFlow (*P* = 0.633). There was a significant difference between the measurement results of the NUCLEOCOUNTER and ANDROVISION with Leja chamber (*P* < 0.0001) and the ANDROVISION eFlow and the Leja chamber as well (*P* < 0.0001). The descriptive data of the sperm concentration measurements are displayed in Table 2. The mean number of sperm per analysis field in the CASA systems was 283.6 ± 53.2 for the eFlow and 173.1 ± 54.0 for the Leja chamber.

**Table 2:** Semen concentration measurement results (mean, SD, minimum and maximum) from 109 diluted boar sperm samples. Mean values differ significantly depending on measurement method (^a,b^ *P* < 0.0001).

Numbers	Nucleocounter (sperm/mL)	Androvision eFlow (sperm/mL)	Androvision with Leja (sperm/mL)
Mean	24.1 × 10^6 a^	23.8 × 10^6 a^	21.6 × 10^6 b^
SD	4.6 × 10^6^	4.6 × 10^6^	6.2 × 10^6^
Min	17.1 × 10^6^	16.3 × 10^6^	9.9 × 10^6^
Max	41.2 × 10^6^	39.7 × 10^6^	44.2 × 10^6^

## Discussion

In order to test the sperm concentration measurement with ANDROVISION eFlow, this study compared different methods for concentration analysis of raw (experiment 1) and diluted boar semen (experiment 2) in split-samples. It is essential to have accurate and precise results for any measurement system. The correctness or accuracy of measurement results can be evaluated by testing the concordance with results of a reference method for a particular parameter in a split-sample approach. We chose the NUCLEOCOUNTER SP-100 as a reference, as the NUCLEOCOUNTER has proven its accuracy and preciseness in numerous examinations for determination of sperm concentration in several species, including porcine^7,9,22^.

Sperm concentration determination of raw semen with ANDROVISION eFlow resulted in a high degree of concordance with the NUCLEOCOUNTER. The mean of 185 sperm concentration measurements in split-samples, only showed a minor, non-significant difference (370.6 × 10^6^ compared to 366.9 × 10^6^ sperm/mL) in all measurement results. The deviation was not systematic, when compared to the reference method. In such a case, the absolute values of the two measurement methods can be considered concordant. In addition, Lin’s Concordance Correlation Coefficient showed a value of 0.955. In contrast to correlative statistics, the CCC does not only test for a linear relationship of measurements from two methods, but also takes the absolute values and their agreement between methods into account^18^. This concept of agreement to describe the relationship between methods is important, when testing method agreements for certain measurement parameters^4,23^. Comparing the mean values of the sperm concentration in diluted semen (experiment 2) for each method demonstrated that the ANDROVISION eFlow measurements in diluted semen did not significantly differ from the values of the reference method (CCC = 0.94). According to Koch and Spörl (2007)^21^, a CCC of 0.94 corresponds to nearly complete concordance. This finding is in accordance with the analysis of raw semen in experiment 1. The Bland–Altman plot^20^ confirms a high degree of concordance in the absolute measurement values, as the average of differences in sperm concentration is 3.62 × 10^6^ sperm/mL and 95.1% of all measurements lie within the mean ± 1.96 SD. The 95% confidence interval of the mean includes the x-axis, confirming the lack of a systematic difference between the two methods^24^. Based on these data, the NUCLEOCOUNTER and ANDROVISION eFlow could be used interchangeably for determining sperm concentration of raw semen samples in this trial.

However, other reports on the accuracy of CASA for sperm concentration measurement have been inconsistent and sparse for boar semen evaluation. In a report with human sperm, comparable results were only obtained in the middle range of all sample concentrations and errors were randomly distributed; often only one third or less of cases agreed within 10%^25^. Correlations of equine sperm concentration estimates obtained with the NUCLEOCOUNTER and those obtained with CASA using Leja 10, 12 and 20 µm slides were low to moderate (*r* = 0.34–0.74), but means were not significantly different^26^. In a comparison of several methods for boar sperm concentration evaluation, CASA results (tested systems: SPERMVISION; IVOS ULTIMATE) differed between − 21.5% and + 41.0% from hemocytometric measurements, which was used as the reference method. The deviation from the hemocytometer in this report was dependent on the absolute sperm concentration (150 × 10^6^, 400 × 10^6^, and 1,100 × 10^6^) and was lowest in the mid-range (400 × 10^6^) of sperm concentrations^9^. Compared to these numbers, the method agreement in experiment 1 and 2 show a higher agreement for both used CASA counting chambers.

Possible reasons for this inconsistency of CASA results in other research reports could lie in the differences in the used hardware (e.g. microscope, camera) and in the CASA software itself. Hardware and software significantly influence the correct detection of sperm cells and especially the capability to properly exclude non-sperm particles from the calculation. Non-sperm particles, as well as agglutinations can influence the sperm concentration result of a CASA system^4^. Other factors may influence the measurement result as well. Douglas-Hamilton et al*.*^27^ described the Segre-Silberberg (SS) effect that occurs in capillary-loaded slides, like many disposable counting chambers. The SS effect is a not uniform particle distribution during influx of suspensions, like diluted semen. The SS effect causes a systematically lower sperm count in these chambers, as the sperm cells accumulate in the Segre-Silberberg planes and will gather at the outlet of the counting chamber, outside the analysis area for CASA. The SS effect can be corrected with a fixed factor that is multiplied with the sperm count of the CASA measurement. Good numerical agreement for sperm concentration with a high degree of correlation (*r*^2^ = 0.94) was found between CASA and hemacytometer, when the SS effect was corrected^27^. The underlying facts of the SS effect also apply to the ANDROVISION eFlow and Leja chamber, and a correction factor is already implemented in the concentration determination by the manufacturer. Other sources of variation in sperm concentration measurement with CASA can be training of technicians, used volumes and pipetting errors during sample preparation, as well as pipetting accuracy^13,28^. Furthermore, the type of used chamber can influence the concentration analysis. Peng et al*.*^14^ found significant differences between diverse chamber types even in analysis of the concentration of latex beads.

The precision of measurement devices can be described by the variation of results of repeated measurement. The coefficient of variation (CV) is usually the parameter of choice to describe the precision of a measurement device or method. With sperm concentration evaluation, depending on the measurement method used, CVs as low as 2.7% can be reached with a flow cytometer and a CV of 3.1% was demonstrated with a NUCLEOCOUNTER^9,29^. Sperm concentration measurement with a CASA resulted in a CV in a range of 5.3%^7^ to 26%^13^ when using disposable counting chambers with a chamber height of 20 µm to evaluate raw boar ejaculates. The results presented here, show a CV of 1.6 ± 1.3%, 3.6 ± 3.6% and 7.3 ± 6.3% for the NUCLEOCOUNTER, ANDROVISION eFlow and ANDROVISION with the Leja disposable counting chamber for diluted semen, respectively. Although these values differ significantly, all CVs are comparatively low in general and are on a level similar as in the aforementioned reports. One reason for this could be the fact that the samples for preciseness were obtained from diluted boar semen and, with exception of the NUCLEOCOUNTER, were analyzed without further pre-dilution. This can prevent variances that are based in pre-dilution and pipetting errors. The CV of eFlow measurements was lower than when using Leja chambers. The possible reason for this includes the higher degree of confirmation of the chamber height of the eFlow with the required value of 30 µm. The chamber height of any counting chamber, which is used for CASA, is included in the calculation of the sperm concentrations and variations in this value will result in variations of the results.

The chamber height of ANDROVISION eFlow is confirmed daily with an internal control module to increase the precision. This control process assures a maximal deviation of ± 10% from the nominal chamber height of 30 µm in the large gap of the eFlow chamber. However, the chamber height of disposable counting chambers may vary depending on production batch. According to the certificates of analysis, the Leja chamber used in this test, allows a median chamber height that diverts up to 10% from the nominal value with a variation of up to 2% from this value^30^. The systematic offset of the measurement results with the Leja chamber may be based on these allowed tolerances and results in a lower CCC of 0.66 between results obtained with the ANDROVISION Leja and the NUCLEOCOUNTER.

As the sample preparation protocol of ANDROVISION eFlow uses higher sample volumes (500 µL) and an automatic fluid management to fill the actual counting chamber, the human influence and error is minimized. Training status of technicians in general is an important factor for the accuracy of sperm concentration measurements. Ehlers et al*.*^13^ could show that the training of laboratory technicians with an e-learning software could decrease the CV of repeated CASA sperm concentration measurements from > 25 to 12%. The higher degree of automation with the eFlow and its fluid management system probably aids with the higher degree of precision and makes it a more accurate and precise measurement system for boar sperm concentration, resulting in a CCC of 0.94 with NUCLEOCOUNTER measurements with diluted semen in experiment 2 for all samples. Figure 5 further confirms a high agreement of these two measurement methods in low and high sperm concentrations, compared to the measurements with ANDROVISION Leja.

However, the production of uniform semen doses is a process of several steps that may include several dilution steps^16^, downtimes during processing with sperm sedimentation and filling of the semen doses^31^. All these steps add up on the variation of sperm number per semen tube; it is therefore important to control each step. In conclusion, the presented results show that NUCLEOCOUNTER and ANDROVISION eFlow are comparable tools to measure the concentration of raw and diluted boar semen. In comparison to ANDROVISION with Leja chambers, the concentration analyses of diluted semen with ANDROVISION eFlow and NUCLEOCOUNTER show a higher repeatability and a higher concordance between the methods.

## Data Availability

The datasets used and/or analyzed during the current study available from the corresponding author on reasonable request.

## References

[1] Schulze M, Nitsche-Melkus E, Jakop U, Jung M, Waberski D (2019). New trends in production management in European pig AI centers. Theriogenology.

[2] Waberski D, Riesenbeck A, Schulze M, Weitze KF, Johnson L (2019). Application of preserved boar semen for artificial insemination: Past, present and future challenges. Theriogenology.

[3] Robinson JAB, Buhr MM (2005). Impact of genetic selection on management of boar replacement. Theriogenology.

[4] Brito LFC (2016). Andrology laboratory review: Evaluation of sperm concentration. Theriogenology.

[5] Anzar M, Kroetsch T, Buhr MM (2009). Comparison of different methods for assessment of sperm concentration and membrane integrity with bull semen. J. Androl..

[6] Jung M, Rüdiger K, Schulze M (2015). *In vitro* measures for assessing boar semen fertility. Reprod. Domest. Anim..

[7] Camus A, Camugli S, Lévêque C, Schmitt E, Staub C (2011). Is photometry an accurate and reliable method to assess boar semen concentration?. Theriogenology.

[8] Brito, L.F.C. *et al.* NAAB-CSS semen quality control program minimum guidelines. (2012).

[9] Hansen C (2006). Comparison of FACSCount AF system, improved Neubauer hemocytometer, Corning 254 photometer, SpermVision, UltiMate and NucleoCounter SP-100 for determination of sperm concentration of boar semen. Theriogenology.

[10] Christensen P, Knudsen DB, Wachmann H, Madsen MT (2004). Quality control in boar semen production by use of the FACSCount AF system. Theriogenology.

[11] Amann RP (2014). Computer-assisted sperm analysis (CASA): Capabilities and potential developments. Theriogenology.

[12] Coetzee K, Menkveld R (2001). Validation of a new disposable counting chamber. Arch. Androl..

[13] Ehlers J, Behr M, Bollwein H, Beyerbach M, Waberski D (2011). Standardization of computer-assisted semen analysis using an e-learning application. Theriogenology.

[14] Peng N, Zou X, Li L (2015). Comparison of different counting chambers using a computer-assisted semen analyzer. Syst. Biol. Reprod. Med..

[15] Gączarzewicz D (2015). Influence of chamber type integrated with computer-assisted semen analysis (CASA) system on the results of boar semen evaluation. Pol. J. Vet. Sci..

[16] Schulze M, Kuster C, Schäfer J, Jung M, Grossfeld R (2018). Effect of production management on semen quality during long-term storage in different European boar studs. Anim. Reprod. Sci..

[17] R Core Team. A language and environment for statistical computing. Vienna, Austria: R Foundation for Statistical Computing (2020). https://www.R-project.org/.

[18] Lin LI (1989). A concordance correlation coefficient to evaluate reproducibility. Biometrics.

[19] Stevenson, M. *et al.* Package ‘epiR’. Tools for the analysis of epidemiological data (2022).

[20] Bland J, Altman DG (1986). Statistical methods for assessing agreement between two methods of clinical measurement. Lancet.

[21] Koch R, Spörl E (2007). Statistische verfahren zum vergleich zweier messmethoden und zur kalibrierung: Konkordanz-, korrelations- und regressionsanalyse am beispiel der augeninnendruckmessung. Klin. Monbl. Augenheilkd..

[22] Danish Agriculture & Food Council. *National Commitee for pig production guidelines for AI stations: Semen Preservation and Health Control* (2005).

[23] Watson PF, Petrie A (2010). Method agreement analysis: A review of correct methodology. Theriogenology.

[24] Giavarina D (2015). Understanding bland altman analysis. Biochem. Medica.

[25] Mortimer D, Aitken R, Mortimer S, Pacey A (1995). Workshop report: Clinical CASA–the quest for consensus. Reprod. Fertil. Dev..

[26] Hoogewijs MK (2012). Influence of counting chamber type on CASA outcomes of equine semen analysis. Equine Vet. J..

[27] Douglas-Hamilton DH, Smith NG, Kuster CE, Vermeiden JPW, Althouse GC (2005). Capillary-loaded particle fluid dynamics: Effect on estimation of sperm concentration. J. Androl..

[28] Broekhuijse MLWJ, Šoštarić E, Feitsma H, Gadella BM (2012). Application of computer-assisted semen analysis to explain variations in pig fertility. J. Anim. Sci..

[29] Hansen C (2002). Validation of the FACSCount AF system for determination of sperm concentration in boar semen. Reprod. Domest. Anim..

[30] Leja Products. *Certificate of Analysis (CoA)* (2021).

[31] Schulze M (2017). Impact of different dilution techniques on boar sperm quality and sperm distribution of the extended ejaculate. Anim. Reprod. Sci..

